# Graphlet-orbit Transitions (GoT): A fingerprint for temporal network comparison

**DOI:** 10.1371/journal.pone.0205497

**Published:** 2018-10-18

**Authors:** David Aparício, Pedro Ribeiro, Fernando Silva

**Affiliations:** CRACS and INESC-TEC, Faculdade de Ciências, Universidade do Porto, R. Campo Alegre, 1021, 4169-007 Porto, Portugal; University of Aveiro, NEW ZEALAND

## Abstract

Given a set of temporal networks, from different domains and with different sizes, how can we compare them? Can we identify evolutionary patterns that are both (i) characteristic and (ii) meaningful? We address these challenges by introducing a novel temporal and topological network fingerprint named Graphlet-orbit Transitions (GoT). We demonstrate that GoT provides very rich and interpretable network characterizations. Our work puts forward an extension of graphlets and uses the notion of orbits to encapsulate the roles of nodes in each subgraph. We build a transition matrix that keeps track of the temporal trajectory of nodes in terms of their orbits, therefore describing their evolution. We also introduce a metric (OTA) to compare two networks when considering these matrices. Our experiments show that networks representing similar systems have characteristic orbit transitions. GoT correctly groups synthetic networks pertaining to well-known graph models more accurately than competing static and dynamic state-of-the-art approaches by over 30%. Furthermore, our tests on real-world networks show that GoT produces highly interpretable results, which we use to provide insight into characteristic orbit transitions.

## Introduction

Networks are widely used to model real-world systems as a way to uncover their topological features [1]. Most of these systems are not static; they exhibit a dynamic nature that can only be captured and truly understood by taking into account the network’s temporal evolution [2]. Consider for instance a co-authorship network, where nodes are authors and edges represent joint publications. By narrowing our focus to static network snapshots we cannot answer relevant questions such as: how stable are connections over time? How is collaboration emerging and dissolving? How did we get to the current state of the network? Can we predict how the network will look like in the future?

One very powerful technique to uncover the underlying structure of a network is to decompose it into smaller components, namely subgraphs. Local network metrics such as network motifs [3] and graphlets [4] incorporate subgraph information to create rich topological metrics that have been successfully applied in many domains. For instance, motif analysis has identified the feed-forward loop as a recurring and crucial functional subgraph pattern in many real biological networks, such as gene regulation and metabolic networks [5, 6], and it was able to identify and separate different families of networks [7]. Another example is the graphlet-degree-agreement, which incorporates the notion of *orbits* (the position of nodes inside each subgraph) and has been used for both network comparison and model fitting, showing that protein-protein interaction networks are more akin to geometric graphs than to traditional scale-free models [4]. These subgraph-based metrics were initially proposed for static networks, thus disregarding temporal information. A temporal extension for graphlets was put forward by Faisal et al. [8]; however, their approach summarizes each temporal snapshot, without offering a real inter-snapshot relation. Another work by Hulovatyy et al. [9] provides a clear inter-snapshot evolution, but they only allow for a single event (i.e., temporal edge) at each snapshot (i.e. just one edge addition between two nodes), therefore limiting the scope of possible graphlet transitions. Our method differs from these because our transition matrix establishes direct relations between snapshots, and we allow for any number of edge additions or removals in each snapshot, aiming for a broader and fully general set of possible transitions between two consecutive snapshots.

In this work we propose graphlet-orbit transitions (GoT) as a framework for characterizing and comparing evolving networks. Our method incorporates the rich topological information provided by subgraphs and extends it to the temporal domain. Orbit-transition matrix encapsulate not only how subgraphs are changing but also how the roles of the nodes themselves are evolving, leading to a more detailed fingerprint of the network. We also introduce the orbit-transition-agreement metric (OTA) as a suitable way of comparing transition matrices of heterogeneous networks.

Next we underline our main contributions:

- **Effectiveness:** GoT achieves over 30% higher precision (AUPR) on a set of well-known network models than other subgraph-based methods. On real data it produces groupings that match pre-determined categories better than competing approaches.
- **Interpretability:** Results produced by GoT are very easy to visualize (i.e. analyze specific transition frequencies between orbits). Therefore, GoT can be used as an interpretable temporal network fingerprint.
- **Generability:** Our method is used to compare heterogeneous networks from different domains and of different sizes. Furthermore, GoT is general and easily extensible to directed and multilayered networks, but these extensions are demanding in terms of storage and execution.

The remainder of this paper is organized as follows. First, we present related work on network comparison with a focus on subgraph-based metrics, both for static and temporal networks. Next, we introduce necessary graph terminology. Our proposed methodology for temporal network comparison is then presented. Finally, various metrics are used to compare and group (i) a set of synthetic data generated using different random-graph models and (ii) a set of real-world data pertaining to different classes, respectively.

## Related work

For a general overview of temporal networks we refer the reader to the survey by Holme and Saramäki [2], and for an overview of existing temporal network metrics we refer to the survey by Nicosia et al. [10].

In this work, our focus is on network comparison, which is a crucial and very useful task. For example, if the properties of a given network are well known, it allows for knowledge transfer to similar networks [11]. Global metrics such as the degree distribution, characteristic path length and clustering coefficient give an idea of the structure of the networks and can be used to compare them. For instance, social networks tend to have a higher clustering coefficient and a smaller characteristic path length than spatial networks. However, these simple metrics are often not expressive enough, and subgraph-based metrics offer a much richer topological characterization.

While our focus here is temporal network comparison using subgraph-based metrics, we should note that pattern discovery on temporal networks is a much broader field. Shah et al. [12] propose an algorithm that concisely summarizes temporal networks by their characteristic temporal subnetworks. Similarly to their work, we also aim for interpretability, but we do graph comparison instead of graph summarization and our method does not require a null model to assess how a certain interesting pattern deviates from randomness. Yu et al. [13] put forward a matrix factorization method that characterizes the correlations of network’s edges as a function of time. Their representation builds a dynamic profile of the network that can be used to predict future states. Here we do not specifically target link prediction; our graphlet-orbit transitions could possibly be used for the task but that is out of the scope of this work. Another task related with both network comparison and network visualization is network condensation [14]; its aim is to reduce the size of the temporal network significantly without much loss of information. Here we aim for interpretability but we do not address the problem of network condensation directly.

On the remainder of this section we give an overview of subgraph-based network metrics, discuss their usefulness and drawbacks, and pinpoint the advantages of our proposed extension when compared to them.

### Static subgraphs

Network motif fingerprints [3] and graphlet-based metrics [4] have been widely used for network comparison. Motifs are overrepresented subgraphs that appear in larger numbers than expected, while graphlet degree distributions can be regarded as an extension of the node degree concept. Both approaches need to compute subgraph frequencies, which is a computationally very expensive task. Even just knowing if one subgraph appears or not on another network is already an NP-Complete problem [15]. Because of this, typically one uses only small subgraphs, but their frequencies already provide very rich characterizations. For instance, Milo et al. [7] compared network motifs with three and four nodes of four superfamilies: sensory networks, hyperlink networks, social networks and linguistic networks. By comparing motif significances they were able to correctly cluster all four superfamilies. Similar studies have been carried out to classify metabolic networks [6], co-authorship networks [16] or articles [17]. Another possibility is to, instead of directly comparing two real networks, compare a network with graph models. Przulj [4] showed that protein-protein interaction networks were more accurately described as random geometric graphs rather than as purely random or scale-free networks. Therefore, motifs and graphlets have been successfully used to compare static networks. However, metrics such as these disregard temporal information which can be crucial for a better understanding of network topology and function.

### Temporal subgraphs

There are several approaches that incorporate the temporal evolution of subgraphs to study and characterize networks. Given the computationally demanding nature of the involved computations, very small or very specific classes of subgraphs are typically used. One example of this are triangles (cliques with three nodes, that is, fully connected sets of three nodes), which are meaningful for many applications since they are the simplest communities. Buriol et al. [18] and Pavan et al. [19] put forward a method to extract approximate and exact counts of all triangles in graph streaming environments. Finocchi et al. [20] proposed an algorithm to count cliques for sizes slightly larger than 3. Instead of triangles, Aliakbarpour et al. [21] focused on star shaped graphs. Our approach differs from these because we support any type of isomorphic subgraphs.

Kovanen et al. [22] presented an extension of network motifs for temporal networks and studied them on a phone call network. Their proposed temporal motifs have at most three events and a varying number of nodes. A similar approach for graphlets was put forward by Hulovatyy et al. [9], without a set limit on either the size of the graphlets nor the total number of events, but which only allows for one event at a time (i.e. their graphlets do not capture instances where two edges, or more, are added in the same snapshot). As a consequence, their method does not directly capture situations where several events occur at the same time, like when a loosely connected subgraph immediately becomes a clique or near-clique. By contrast, our approach supports any number of edge removals or additions, allowing for the analysis of networks that intrinsically have multiple events occurring at the same time.

Martin et al. [23] proposed a metric to evaluate network similarity based on how their triplets are evolving over time. Their metric is based on the loss or gain of edges from one state to the next. Our method differs from theirs since they do not differentiate by pair-wise graphlet transitions but only by increase or decrease of total edges between states (i.e. different pair-wise transitions are not differentiated as long as they affect the same number of edges). The approach by Doroud et al. [24] is more similar to our own since they enumerate all transitions between 3-node directed subgraphs in network snapshots. That information is used in order to estimate the probability of a given transition in a social network and predict network changes. Kim et al. [25] also count all 3-node directed subgraphs to assess which motifs are present in different states of developing gene networks in different regions. These approaches are however limited to 3-node subgraphs and do not consider the roles of the individual nodes, that is, the orbits.

Faisal and Milenkovic [8] integrate graphlet frequency distributions on the analysis of temporal biological networks, but they only look at the global distribution in each snapshot, without offering the possibility to observe how each individual connected set of nodes is evolving. By contrast, we provide a direct transition matrix.

Another approach is followed by Jin et al. [26]; they introduce the notion of trend motifs geared towards weighted networks, trying to capture increasing or decreasing trends in the weights of specific sets of nodes inducing certain subgraphs. Therefore, their approach is only applicable to weighted networks, not identifying edge appearance and disappearance as in our case.

## Preliminaries

### Graph terminology

A *graph* (or *network*) *G* is comprised of a set *V*(*G*) of *vertices* (or *nodes*) and a set *E*(*G*) of *edges*. A *k*-graph is a graph with *k* nodes. Nodes represent entities and edges correspond to relationships between them. Edges are represented as pairs of vertices of the form (*u*, *v*), where *u*, *v* ∈ *V*(*N*). In *directed* graphs, edges (*u*, *v*) are *ordered pairs* (translated to “*u goes to v*”) whereas in *undirected* graphs there is no order since the nodes are always reciprocally connected.

A *subgraph occurrence S*_*k*_ in *G* is a *k*-graph where *V*(*S*_*k*_) ⊆ *V*(*G*) and *E*(*S*_*k*_) ⊆ *E*(*G*). A subgraph occurrence is *induced* if ∀*u*, *v* ∈ *V*(*S*_*k*_): (*u*, *v*) ∈ *E*(*S*_*k*_) iff (*u*, *v*) ∈ *E*(*G*). Two subgraph occurrences are said to be *isomorphic* if it is possible to obtain one from the other just by changing the node labels without affecting their topology. The general task of evaluating if two graphs are isomorphic is called the graph isomorphism problem [27]. If two subgraphs occurrences *S*_*k*_ and $S_{k}^{\prime }$ are isomorphic, they are occurrences of the same *subgraph G*_*k*_ (i.e., canonical class [28]). The *frequency Fr*(*G*_*k*_, *G*) is the number of occurrences of *G*_*k*_ in *G*.

### Subgraph census

Graphlets and network motifs have at their core the task of computing subgraph frequencies. This is known as the general subgraph census problem [29]:

**Definition 1 (Subgraph census)** *Given a set* $G_{k}$ *of non-isomorphic k-subgraphs and a graph G, determine the* ***frequency of all induced occurrences*** *of the subgraphs* $G_{k}\in G_{k}$ *in G. Two occurrences are considered different if they have at least one node or edge that they do not share. Other nodes and edges can overlap*.

### Static network motifs

Network motif analysis has two steps: first, subgraph census is performed on graph *G*, and second, motif significance is assessed on a null model [3]. Numerous null models can be used, such as the one proposed in [3] which generates a set R(G) of randomized networks that keep *G*’s degree sequence. A subgraph census is then performed on each R∈R(G). The average frequency of a subgraph *G*_*k*_ on the randomized networks is represented by $<Fr(G_{k},R\left(G\right))>$, and *G*_*k*_ is considered a network motif if it appears with a significantly higher frequency in *G* than in R(G). Motif scores, represented by *δ*_*k*,*G*_, are computed for each subgraph *G*_*k*_ (Eq 1). As was proposed in [7], motif scores are normalized, represented by Δ_*k*,*G*_ (Eq 2).

$$\begin{array}{c} \delta_{k,G}=\frac{Fr\left(G_{k},G\right)\;-<Fr\left(G_{k},R\left(G\right)\right)>}{Fr\left(G_{k},G\right)\;+<Fr\left(G_{k},R\left(G\right)\right)>} \end{array}$$ (1)

$$\begin{array}{c} \Delta_{k,G}=\frac{\delta_{k,G}}{\sqrt{\sum {\left(\delta_{k,G}\right)}^{2}}} \end{array}$$ (2)

The motif-fingerprint of *G* is a vector containing all Δ_*k*,*G*_.

### Static graphlets

Graphlets [4] are small induced non-isomorphic subgraphs that differentiate nodes according to their subgraph positions—or their *orbits*. In a graphlet *G*, the set of isomorphisms of *G* into itself comprises its set of *automorphisms*. Two vertices *u* and *v* are said to be equivalent (meaning *“in the same orbit”*) when there exists some automorphism that maps *u* into *v*.

**Fig 1:**
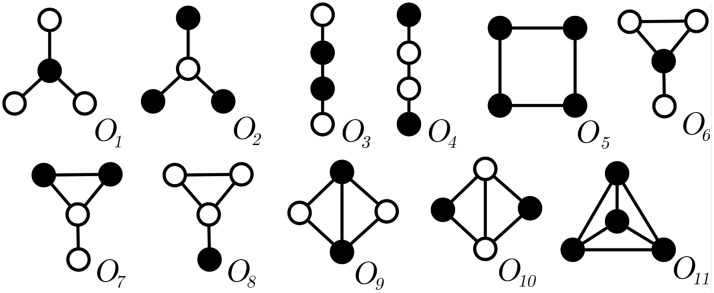
Set of all 11 graphlet-orbits of subgraphs with 4 nodes: $uO_{4}$. Black nodes belong to the same graphlet-orbit.

Fig 1 presents all undirected graphlet-orbits with 4 nodes. For instance, the single node at the center of a *star* is topologically different from a leaf-node, whereas leaf-nodes are structurally equivalent. Therefore, a *4-star* has only two orbits: a *center-orbit O*_1_ which a single node inhabits, and a *leaf-orbit O*_2_ where the remaining 3 nodes are at. Graphlets can be either undirected [4] or directed subgraphs [30]. Notation $uG_{k}$ is adopted for the set of all undirected graphlets with *k* nodes, and $dG_{k}$ for directed ones. The set of all orbits of $uG_{k}$ is expressed as $uO_{k}$, and $dO_{k}$ is used for directed graphlets. Prefixes *d* and *u* are suppressed whenever concepts are applicable to both directed and undirected graphlets.

**Fig 2:**
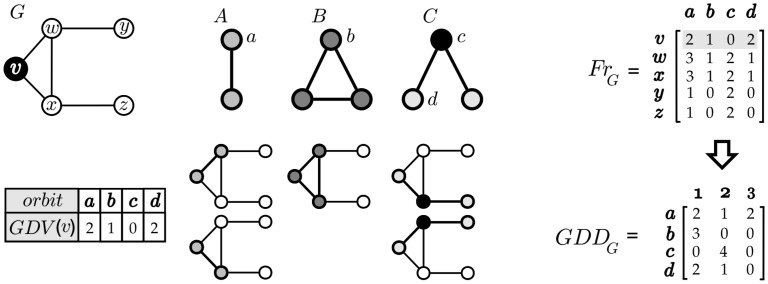
*GDV*(*v*) obtained by enumerating all undirected graphlet-orbits of sizes 2 and 3 (*A*, *B* and *C*) touching *v*, and resulting *Fr*_*G*_ and *GDD*_*G*_ matrices for the complete subgraph census (*GDV*(*v*) is highlighted in gray in *Fr*_*G*_).

The graphlet-degree distribution is an extension of the node degree-distribution, and both can be used for network comparison. The degree distribution of a given graph *G* is obtained by counting ∀*u* ∈ *V*(*G*) how many direct connections *u* has. This task produces a vector of size *n* = |*V*(*G*)| containing the degrees of each *u* ∈ *V*(*G*) which is transformed into a node-degree-distribution vector (or *NDD*_*G*_, for short) of size *m*, where *m* is the maximum degree, and *NDD*_*G*,*p*_ is the number of nodes that have degree *p*. Notice from Fig 2 that the node-degree is essentially equivalent to orbit *a* (a two node subgraph): the node-degrees of each node *n* correspond to the first column of *Fr*_*G*_ and that *NDD*_*G*_ is simply the first line of *GDD*_*G*_.

Graphlet-degree-distributions (*GDD*_*G*_) generalize the concept of *NDD*_*G*_ for subgraphs bigger than the degree (i.e., subgraphs with more than two nodes). To compute the graphlet degree distribution it is necessary to count ∀*u* ∈ *V*(*G*) how many times *u* appears in some orbit j∈O and repeat this process for the total |O| orbits, resulting in a graphlet degree vector *GDV*(*u*). A matrix *F*_*r*_(*G*) of *n* × *m* positions is obtained by joining the *GDV*s of all *n* nodes where each row of *Fr*(*G*) is *GDV*(*u*), *u* ∈ *V*(*G*) and each position *fr*_*u*, *j*_ is the number of times that node *u* appears in orbit *j*. Matrix *GDD*_*G*_ is obtained directly from *Fr*_*G*_, where $GDD_{G}^{j,p}$ is the number of nodes that appear *p* times in orbit *j*. This task is more formally defined in Definition 2 and an example of this process is given in Fig 2. For instance, node *v* has degree 2 (orbit *a*), appears once in a triangle (orbit *b*), and appears in 2 chains always in its periphery (orbit *d*).

**Definition 2 (Graphlet-orbit frequency computation)** *Given a set* $G_{s}$ *of non-isomorphic subgraphs of size s and a graph G, determine the number of times fr*_*i*,*j*_ *that each node i* ∈ *V*(*G*) *appears in all the orbits* $j\in O_{s}$. *All occurrences are induced. Two occurrences are considered different if they have at least one node or edge that they do not share. Other nodes and edges can overlap*.

As suggested by Pržulj [4], a GDD matrix is normalized with respect to its total area (i.e., the sum of all $GDD_{G}^{j,p}$), before being used for comparison. The normalized values are represented below as $n_{G}^{j,p}$. Two networks *G* and *H* are compared by computing the differences between their respective normalized *GDD* matrices. One possibility to compare the two matrices is to use the arithmetic mean GDD-agreement (*GDA*) introduced in [4]: m6) the GDA is obtained individually for each orbit *j* (Eq 3) and then the mean GDA is computed (Eq 4), m7) ranging from 0 to 1. High *GDA*(*G*, *H*) means that *G* and *H* are topologically similar. Note in Eq 3 that, in practice, *p* is never infinite because, in real graphs, the number of nodes that appear in a given orbit is always finite.

$$\begin{array}{c} GDA{\left(G,H\right)}^{j}=1-\frac{1}{\sqrt{2}}\sqrt{\left(\sum_{p=1}^{\;+\infty }{\left[n_{G}^{j,p}-n_{H}^{j,p}\right]}^{2}\right)} \end{array}$$ (3)

$$\begin{array}{c} GDA\left(G,H\right)=\frac{1}{m}\sum_{j=0}^{m}GDA{\left(G,H\right)}^{j} \end{array}$$ (4)

### Temporal networks

Temporal networks used throughout this paper consist of *s* consecutive snapshots of a global network *N*, where *N* belongs to a set of temporal networks N. The set of all snapshots of *N* is referred to as $S_{N}$. An edge (*u*, *v*) exists in snapshot $S_{N,i}\in S_{N}$ if nodes *u* and *v* are connected in the interval [*I*_*N*_ + *ρ* × *i*, *I*_*N*_ + *ρ* × (*i* + 1)[, where *I*_*N*_ is the starting time of the network and *ρ* is the time-interval. A temporal edge is also referred to as an *event*. Parameters *ρ* and *s* depend on the network; for instance, in scientific co-authorship networks one or two years are the more suitable value for *ρ*, while in conference interaction networks *ρ* is a few hours or a couple of days. The number of snapshots $|S_{N}|$ depends on the amount of available data. Networks can gain (or lose) new edges (or new nodes) from *S*_*i*_ to *S*_*i*+1_.

## Computing temporal network similarity using Graphlet-orbit Transitions

In this section we describe our method and specify how it is used to measure temporal network similarity. Temporal network similarity assumes that there is a set of temporal patterns used as features; in our case those features are graphlet-orbit transitions (GoT). A metric is also necessary to compare the networks’ feature space; for this purpose we developed orbit-transition agreement (OTA).

**Definition 3 (Temporal Network Similarity)** *Given two temporal networks G and H*, ***compute how similar they are. Their similarity is given by how similar their temporal patterns are***.

**Definition 4 (GoT Similarity)** *Given two temporal networks G and H and a set of graphlets*, ***compute their graphlet-orbit transition matrices*** $T_{x,y}\left(G\right)$ *and* $T_{x,y}\left(H\right)$, *respective to each network*. ***Their similarity is given by the orbit-transition agreement* (OTA) *of their matrices***.

### Graphlet-orbit Transitions (GoT)

In essence, our method performs graphlet-orbit frequency computation (as stated in Definition 2) for each snapshot of a given temporal network. Our aim is to analyze how the roles of nodes are evolving between snapshots. Only connected graphlets are taken into account because our focus is to study how groups evolve and, when a group becomes disconnected, that set of nodes is no longer a group. We should point out that disconnected graphlets would be very useful to analyze group formation, but computing their frequencies would require considering all possible (nk) subsets of nodes, effectively making it only feasible for small networks and very small *k*-graphlets.

Possible algorithms for graphlet-orbit frequency enumeration include [31, 32]; here we use g-tries due to their general applicability and efficiency [33]. G-Tries can be used to store subgraphs of any given size, as long as they fit into main memory, with directed or undirected edges. Their efficiency comes from compressing the search space by taking advantage of common subtopolgies in the input subgraphs. For more details on how g-tries are created and how they are used for graphlet-orbit frequency enumeration we refer the reader to [30, 33].

**Fig 3:**
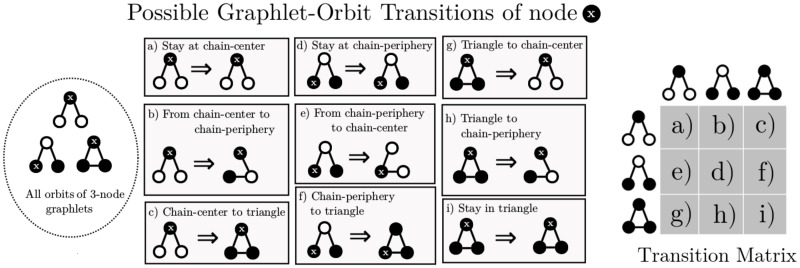
All possible orbit transitions of 3-node undirected graphlets and corresponding orbit-transition matrix. Node *x* is the node being currently considered and black nodes are nodes in the same orbit as *x*.

**Fig 4:**
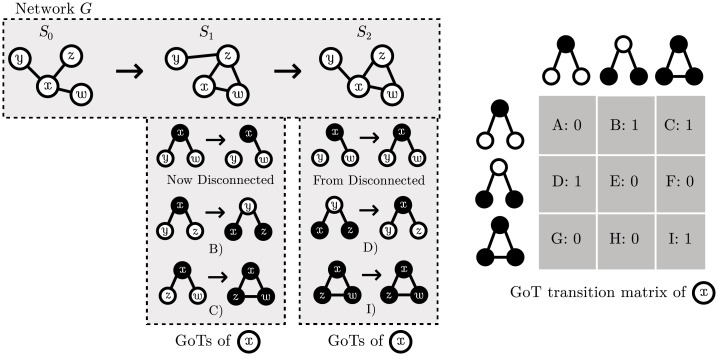
Graphlet-orbit transitions of node *x*. Note that transitions to (and from) disconnected graphlets are not considered.

G-Tries allow the user to customize which subgraphs are enumerated (i.e. only stars, only cliques, etc.). To showcase the general scope of our method we search for all subgraphs of size *k*. Consider the two possible 3-node undirected graphlets, $uG_{3}$, and their respective orbits from Fig 3. The chain-graph has two possible positions—the node can be either at its center or in one of its leaves—while all nodes in a triangle-graph are topologically equivalent. Given those three possible orbits, GoT counts how many times a node *x* changes from one to the other. There are 3 × 3 = 9 possible orbit transitions for $uO_{3}$. A node can remain in its previous orbit, be it a (a) chain-center, (d) chain-periphery or (i) triangle-node; it can go from the chain-center to the chain-periphery (b) or to a triangle-node (c), etc. All possibilities are shown in Fig 3. Fig 4 shows an example of a temporal network and the GoTs of a single node *x*.

Algorithm 1 gives an overview of the enumeration process that builds the transition matrices. In order to obtain the frequencies of these transitions, for each snapshot *t* our method enumerates all *k*-node occurrences {*Node*_1_, *Node*_2_, …, *Node*_*k*_} (lines 3-5) as well as the orbits of each node on that subgraph {*Orbit*_1_, *Orbit*_2_, …, *Orbit*_*k*_} (line 6). The occurrences discovered are pushed into a vector of the form {*Node*_1_, *Node*_2_, …, *Node*_*k*_, *t*, *Orbit*_1_, *Orbit*_2_, …, *Orbit*_*k*_} (line 7). When all occurrences have been enumerated one can simply sort the vector (line 9) and check if two consecutive vector positions contain the same subgraph (but were found in consecutive snapshots) (lines 10-11). As an example, occurrences {5, 8, 10, 12, *t* = 1, *x*, *x*, *y*, *y*} and {5, 8, 10, 12, *t* = 2, *x*, *y*, *x*, *y*} increment graphlet-orbit transitions $T_{x,x}\left(N\right)$, $T_{x,y}\left(N\right)$, $T_{y,x}\left(N\right)$ and $T_{y,y}\left(N\right)$ all by 1 (regardless of what orbits *x* and *y* represent). These transitions are used to build the network’s transition matrix (lines 12-14); they offer rich topological information that can be used for network summarization, Data Mining (e.g. they can be used as features for prediction tasks), network comparison and model fitting. In this work we compare different networks according to their transition matrices. Next we describe our metric for network comparison based on orbit-transition matrices.

**Algorithm 1** Enumerate graphlet-orbit transitions of orbits $O_{k}$ on temporal network *N*.

1: **procedure** enumerateOrbitTransitions(*N*, $O_{k}$)

2:  *Fr* = ∅

3:  **for all** snapshots $S_{N,t}\in S_{N}$ **do**

4:   **while** enumerateSubgraph(*S*_*N*,*t*_, $O_{k}$) finds an occurrence **do** (Def 2, G-Tries)

5:    occurrence: {*Node*_1_, *Node*_2_, …, *Node*_*k*_}

6:    {*Orbit*_1_, *Orbit*_2_, …, *Orbit*_*k*_} = *getOrbits*({*Node*_1_, *Node*_2_, …, *Node*_*k*_})

7:    *Fr*.*append*({*Node*_1_, *Node*_2_, …, *Node*_*k*_, *t*, *Orbit*_1_, *Orbit*_2_, …, *Orbit*_*k*_})

8:   $T_{x,y}\left(N\right):$ fill with zeros

9:   sort(*Fr*)

10:   **for all** consecutive *Occ*_1_, *Occ*_2_ ∈ *Fr* **do**

11:    **if** *Nodes*(*Occ*_1_) == *Nodes*(*Occ*_2_) **then**

12:     **for** *i* ∈ {1, …, *k*} **do**

13:      (*x*, *y*): (*Orbits*(*Occ*_1_)[*i*], *Orbits*(*Occ*_2_)[*i*])

14:      $T_{x,y}\left(N\right)++$

15:   **return** $T_{x,y}\left(N\right)$

### Orbit temporal agreement (OTA)

After enumerating all graphlet-orbit transitions, and having constructed $T_{x,y}\left(N\right)$ matrices for each network *N* of set N, our method computes their topological similarity. For experimental purposes, all orbits of size *k* are enumerated for each network, therefore each $T_{x,y}\left(N\right)$ matrix consist of |O|×|O| transitions. Our approach is based on the arithmetic mean of orbit-transition differences. Matrices $T_{x,y}\left(N\right)$ are normalized before computing orbit-transitions differences in order to reduce bias induced by different network sizes. Normalization is performed by row, as shown in Eq 5. This choice gives the same importance to common and rare orbits. Instead, one could normalize the matrix both by row and column if the scale of the original values is important. We feel that choosing the latter option would disregard differences in rare orbits, which arguably can differentiate networks better than common ones.

$$\begin{array}{c} ntr_{i,j}=\frac{tr_{i,j}}{\sum_{k=1}^{\left|O\right|}tr_{i,k}} \end{array}$$ (5)

The similarity of two networks *N*_1_ and *N*_2_ is given by the average similarity of their graphlet-transition frequency for each graphlet-transition *ntr*_*i*,*j*_. Eq 6 presents this metric, which we name orbit-transition agreement (*OTA*).

$$\begin{array}{c} OTA\left(N_{1},N_{2}\right)=\frac{1}{{\left|O\right|}^{2}}\times \sum_{i=1}^{\left|O\right|}\sum_{j=1}^{\left|O\right|}(1-|ntr_{i,j}^{N_{1}}-ntr_{i,j}^{N_{2}}|) \end{array}$$ (6)

Eq 6 produces an *absolute value of agreement*, i.e., *OTA*(*N*_1_, *N*_2_) is always the same regardless of N. However, for our purposes a *relative value of agreement* is more suitable since we want to compare networks within a specific set. Consider $max(OTA_{N})$ and $min(OTA_{N})$ as the highest and lowest *OTA* between two networks in set N, we normalize the *OTA* matrix to values between 0 and 1 (Eq 7). Using the normalized *nOTA*, the two most similar networks on the set N have *nOTA* = 1, and the two most different have *nOTA* = 0, while the other pairs have a normalized *nOTA* between 0 and 1.

$$\begin{array}{c} nOTA_{N_{1},N_{2}}=\frac{OTA_{N_{1},N_{2}}-min\left(OTA_{N}\right)}{max\left(OTA_{N}\right)-min\left(OTA_{N}\right)} \end{array}$$ (7)

Algorithm 2 shows our overall methodology.

**Algorithm 2** Compute network similarity of set N using *k*-node orbit-transitions

1: **procedure** computeNetworkSimilarity(N, *k*)

2:  $O_{k}:$ generate all *k*-node orbits.

3:  **for all** networks N∈N **do**

4:   $T_{x,y}\left(N\right)$ = enumerateOrbitTransitions(*N*, $O_{k}$)

5:   normalize($T_{x,y}\left(N\right)$)            (Eq 5)

6:  **for all** pairs $\left\{\left(N_{1},N_{2}\right)|N_{1},N_{2}\in N\right\}$ **do**

7:   *OTA*(*N*_1_, *N*_2_) = getOTA ($T_{x,y}\left(N_{1}\right),T_{x,y}\left(N_{2}\right)$)  (Eq 6)

8:  **for all** pairs $\left\{\left(N_{1},N_{2}\right)|N_{1},N_{2}\in N\right\}$ **do**

9:   normalize(*OTA*(*N*_1_, *N*_2_))            (Eq 7)

## Classifying synthetic data

We assess our method’s grouping efficiency on a set of well-known graph models, and compare it against other subgraph-based methods. Our assumption is that an efficient method should report networks from the same model as more topologically alike than networks from different models due to their inherent structure (a similar approach was followed in [9]). All of the following experiments were conducted on an Intel i7-6700 CPU with 4 cores at 3.40 GHz; nevertheless, all programs were executed using a single-thread. Our code was written in C++11 and compiled with gcc 6.3.1 with O3 optimizations, while dynamic graphlets [9] were computed using the executable available at http://www3.nd.edu/~cone/DG/. Network motifs, graphlets and static-temporal graphlet vectors were obtained using our own code, available at http://www.dcc.fc.up.pt/~daparicio/software. The source code for graphlet-orbit transitions computation, as well as the data used for experimental purposes, can be found at http://www.dcc.fc.up.pt/got-wave/ and dx.doi.org/10.17504/protocols.io.tcqeivw.

### Synthetic networks

In order to assess our method’s clustering capabilities we tested it on dynamic versions created by us of three of the most well-known and studied random-graph models: Erdos-Rényi [34], Baràbasi-Albert [35] and Watts-Strogratz [36]. All synthetic networks have 5 snapshots and start with 250 nodes; these values were chosen in order to obtain results from every method in a reasonable time. New nodes and edges arrive in the networks [37], while the network’s density remains stable throughout all snapshots (this behavior was observed in online social networks [38], for instance). New edges are created according to the model’s criteria: either randomly [34], by preferential attachment [35] or through rewiring of past edges [36]. Noise is also injected in some of the networks by having edges randomly deleted: if *P*(*e*^−^) = 0.5, half of the edges from *S*_*N*,*i*_ are removed in *S*_*N*,*i*+1_, whereas if *P*(*e*^−^) = 0, all edges are permanent. Strogratz models control how much rewiring is performed; we use either no rewiring (*β* = 0) to build regular ring-networks or some rewiring (*β* = 0.2) to create small-world networks. From these variables we obtain a total of 6 different graph models (see Table 1), and build 25 instances/networks of each.

**Table 1:** Set of random network models used for evaluation.

	$|S_{N}|$	V(S_N,1_)	V(S_N,i+1_)	$\frac{E}{N^{2}}$	P(e^−^)	P(e^+^)	P(β)
Erdos	5	250	*V*(*S*_*N*,*i*_) + 0.1 × *V*(*S*_*N*,*i*_)	0.01	0.0 0.5	Random	–
Barábasi	5	250	*V*(*S*_*N*,*i*_) + 0.1 × *V*(*S*_*N*,*i*_)	0.01	0.0 0.5	Degree	–
Strogratz	5	250	*V*(*S*_*N*,*i*_) + 0.1 × *V*(*S*_*N*,*i*_)	0.01	–	Ring creation and Rewiring	0.0 0.2

### Methods

In our experiments we assess GoT’s grouping capabilities and compare it to other subgraph-based methods. All methods rely on subgraph census, as defined in Definition 1, therefore an appropriate set of *k*-subgraphs needs to be chosen. Hulovatyy et al. [9] reported that dynamic graphlets with 4-nodes and 6-events achieved the best results for node classification tasks, and that increasing their size did not significantly improve results and greatly increased computational time. Therefore, 4-node and 6-event dynamic graphlets are enumerated and, for results to be directly comparable, 4-node subgraphs are enumerated for every other method. For generability, all possible 4-node subgraphs are enumerated instead of a specific set.

- **Static motifs (SM) –** 4-node subgraphs (network motifs) are enumerated on a single aggregate network, and their *motif score* is evaluated on a set of 100 similar randomized networks (see [7]). Output: a vector of *motif scores* for each network.
- **Static graphlets (SG) –** 4-node graphlet-orbits are enumerated on a single aggregate network (see [4]). Output: a vector of orbit frequencies for each network.
- **Static-temporal graphlets (STG)—** 4-node graphlet-orbits are enumerate on each network snapshot (see [8]). Output: a vector concatenating the orbit frequencies of each network snapshot.
- **Dynamic graphlets (DG) –** 4-node graphlets with at most 5 events are enumerated on the temporal network (see [9]). Output: a vector of dynamic graphlet frequencies.
- **Graphlet-orbit transitions (GoT) –** 4-node graphlet-orbit transitions are enumerated. However, for the methods to be more easily comparable, the OTA is not computed. Output: a matrix of graphlet-orbit transitions.

### Accuracy and speed comparison

For each node, we compute its SM, SG, STG, DG and GoT vectors and use them as the node’s features. For instance, when considering GoT, each node is represented by its graphlet-orbit transitions. The feature vectors over all nodes in a network form a #Nodes × #Features matrix. For two networks being compared, this results in two corresponding matrices with the same number of columns, whose rows are then joined together. Due to high dimensionality and sparsity of the joined matrix, we perform dimensionality reduction on the matrix using principal component analysis, keeping 99% of its variance. Then, we compute the topological similarity between every two nodes from different networks as the Euclidean similarity between the nodes’ PCA-reduced feature vectors.

**Fig 5:**
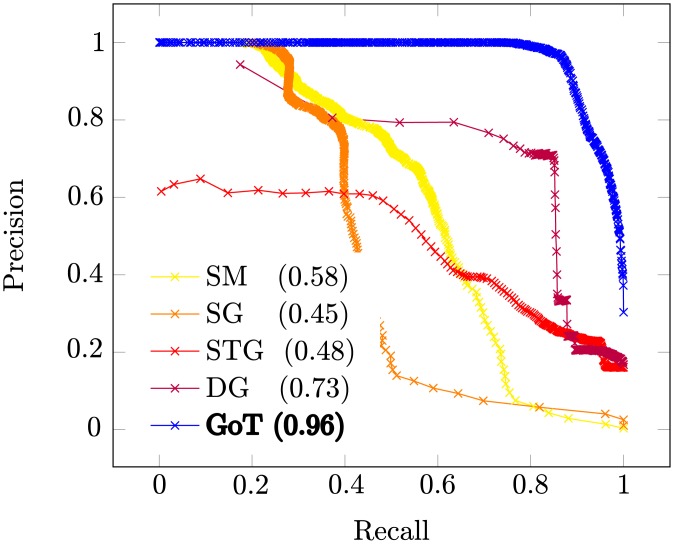
Obtained precision-recall curves on synthetic data (AUPR inside parentheses).

Precision-recall curves (PRCs) were calculated and are presented in Fig 5. In order to compute the PRCs, *ϵ* is initially set as 0 (meaning that the networks are exactly the same according to the metric) and is incremented by *s* = 0.001 at each step until *s* = 1 (the networks are totally distinct). Precision is the fraction of correctly grouped pairs while recall is the fraction of the correctly grouped pairs over all correct ones. The Area Under the Precision-Recall curve (AUPR) evaluates how well the metric groups the networks, and its value is approximated as shown in Eq 8. *Pr*(*k*) is the precision at step *k*, Δ*Rec*(*k*) is the change in recall from steps *k* − 1 to *k* and *N* is the number of steps.

$$\begin{array}{c} AUPR=\sum_{k=1}^{N=1000}Pr\left(k\right)\Delta Rec\left(k\right) \end{array}$$ (8)

Our method (GoT) achieves the highest AUPR and has a gain of ≈ 30% when compared to the second best (DG). STG obtained a higher AUPR than SG, but only by a small fraction, while DG performed significantly better than both, corroborating the results from [9]. Table 2 compares the execution times of the two approaches that achieve highest AUPR: GoT and DG [9]. For Baràbasi networks times are comparable; this is due to the high density of Baràbasi networks that induce a larger number of GoT transitions. In our experiments it is clear that our method is both faster and more accurate than competing approaches on a set of simple and well-known evolving random graph models. For the Strogratz model with no rewiring (*P*(*β*) = 0.0), in particular, our method is over 400 times faster than DG [9]. This high efficiency comes from the data-structure and algorithm that we use, based on g-tries [33].

**Table 2:** Time comparison of our method (GoT) and dynamic graphlets (DG). We show the speed gain of GoT over DG inside parentheses (e.g., 2x means 2 times faster).

	Erdos: *P*(*e*^−^)		Baràbasi: *P*(*e*^−^)		Strogratz: *P*(*β*)	
	0.0	0.5	0.0	0.5	0.0	0.2
*DG*	5.92s	17.84s	5.96s	471.68s	52.44s	33.84s
GoT	0.76s (8x)	0.84s (21x)	4.50s (1.3x)	5.36s (88x)	0.12s (437x)	0.52s (65x)

### Discussion on storage limitations and execution times

Typically subgraph census is only feasible for relatively small networks and small subgraphs. This is due to the number of occurrences, and therefore the resulting execution time, growing exponentially (i) on larger (or denser) networks and (ii) with the size of the subgraphs [33].

Consider Table 3, the number of possible orbits grows exponentially with the number of nodes; this effect is even more pronounced in directed subgraphs. GoT stores all possible graphlet-orbit transitions (or #*Orbits*^2^), further increasing the strain on storage space. Assuming that the frequency of each transition is stored in a 4-byte integer, computing GoT requires 4 × #*Orbits*^2^ bytes of memory. Therefore, ≈ 2GB of RAM are needed when enumerating all 5-node directed GoT, which is feasible in modern PCs. However, enumerating all 6-node directed GoT is impractical. A possible solution to reduce the memory footprint is to remove orbit redundancies [4]. Another option is to avoid generating all possible orbits before enumeration and instead only build their representation during the enumeration phase as they occur in the network [39] since it is reasonable to expect that only a fraction of all possible orbits actually appear in a given network.

**Table 3:** Total number of possible orbits and GoTs per subgraph size *k*.

*k*	$uG_{k}$		$dG_{k}$	
	#Orbits	#GoT	#Orbits	#GoT
3	3	3^2^ = 9	30^2^	900
4	11	121	697	>485k
5	58	3364	>44k	>2B
6	407	>165k	>9M	>81T

Another problem comes from the exponential increase in the number of occurrences as *k* grows. Table 4 shows the average number of occurrences for all 25 networks of three models from Table 1: Erdos and Barabasi with *P*(*E*^−^) = 0 and Strogratz with *P*(*β*) = 0.2. It can also be noted that, despite having the same (a) number of nodes, (b) number of edges and (c) density, the number of occurrences varies greatly. Barabasi networks induce many more subgraph occurrences since they have a much higher cluestering coefficient than both Erdos and Strogratz networks. This quick growth in the number of occurrences makes subgraph census generally only feasible for small networks and small subgraphs. Previous work has extended g-tries to employ both sampling [40] and parallelism [41] to greatly reduce enumeration time, making larger subgraph sizes attainable (*k*> 6). We should note that our method requires a subgraph enumeration algorithm, since it needs not only the subgraph frequencies but also their occurrences, thus very efficient subgraph counting methods such as [32, 42] can not be used. While subgraph enumeration is a very challenging problem, the field is very active and currently it is possible to scale to networks with millions of edges and subgraphs with more than 6 nodes by combining efficient algorithms, parallelism and sampling.

**Table 4:** Average number of *k*-size subgraph occurrences per network.

*k*	#Occurrences		
	Erdos	Barabasi	Strogratz
3	≈ 40k	≈ 175k	≈ 34k
4	≈ 350k	≈ 9M	≈ 270k
5	≈ 3.8M	≈ 450M	≈ 2.6M
6	≈ 45M	≈ 10B	≈ 28M

## Grouping and analyzing real data

In this section we show the effectiveness of our proposed method in (a) grouping a set of real-world temporal networks by predetermined categories and (b) visualizing their characteristics. Therefore, our goals are to assess grouping capabilities but also interpretability. The set of real-world networks N comprises (i) co-authorship, (ii) crime, (iii) e-mail communication, (iv) physical interaction, (v) bipartite, (vi) soccer transfers and (vii) social media friendship networks, as shown on Table 5. Our hypothesis is that networks of the same category have similar topological structure [7, 9], and this is verified by our method.

**Table 5:** Set of temporal networks N grouped by category.

Name	Nodes	Edges	*ρ*	|S|	Source
Authenticus	authors 7k	co-author a paper 120k	1 year	16	Our own.
arXiv hep-ph	authors 2k	co-author a paper 357k	1 year	7	[43]
Minneapolis	streets 454	crime in intersection 12k	3 months	16	[44]
Philadelphia	streets 1k	crime in intersection 10k	3 months	16	[45]
Emails	workers 167	email between workers 83k	1 month	9	[46]
Enron	workers 6k	email between workers 51k	2 months	16	[47]
Gallery	visitors 420	physical interaction 43k	4 days	16	[48]
Conference	visitors 113	physical interaction 21k	12 hours	6	[48]
School	students 327	physical interaction 189k	1 day	5	[49]
Workplace	workers 92	physical interaction 10k	10 days	10	[50]
Escorts	clients + escorts 10k + 7k	hires 51k	3 months	16	[51]
Twitter	users + hashtags 12k + 16k	user tweets hashtag 327k	3 months	16	[52]
Transfers	soccer teams 2k	player transfer 20k	1 year	16	Our own.
Facebook	friends 47k	posts on the other’s wall 877k	3 months	16	[53]

We start by analyzing how networks are evolving over time (*growing* vs.*shrinking*, becoming *more-connected* vs.*less-connected*) as well as some of their global metrics, namely the *average-degree*, the *clustering-coefficient* and the *characteristic path-length*. These metrics are easy to analyze visually and give some temporal information about the networks, but they are not successful when grouping the networks due to their limitations. Static network motif (SM) and graphlet (SG) analyses are also conducted since they capture richer topological information than aforementioned global metrics. We compare the networks’*motif-fingerprints* and *graphlet-degree distributions* for 4-node subgraphs and assess how well the networks are being grouped using these metrics. We assess the clustering capabilities of static graphlet-orbits by computing the graphlet-degree-agreement (*GDA*) for each pair of networks and clustering set N accordingly: networks with high agreement are grouped together. We proceed in a similar fashion for our own graphlet-orbit transitons by computing the orbit-transition-agreement (*OTA*) for each pair of networks. Finally, we show that graphlet-orbit transition matrices offer highly interpretable information which displays both (a) clear differences between networks of different categories and (b) characteristic transitions in networks of the same category.

Here we do not show results for static temporal graphlets (STG) [8] because they did not show significant improvement in our synthetic data (Table 1) and they are harder to visualize than static graphlets. Dynamic graphlets (DG) [9] with 4 nodes and 5 or 6 events were computed in our set of networks N but, for some networks, the method did not output graphlet counts in a manageable time, making it impossible to compare with our method. Table 6 shows a comparison of the execution times between our method (GoT) and dynamic graphlets (DG). All possible 4-node graphlets were enumerated by both methods. Dynamic graphlets have the number of events as an additional parameter; thus, dynamic graphlets with 5 events (DG-5) or 6 events (DG-6) were separately computed. For some of the largest networks from Table 5 neither DG-5 nor DG-6 produced an output in a reasonable time (we allowed it to run for over a week). For the networks that both GoT and DG finished their computation it is clear that DG is much more computationally heavy. Furthermore, growing the number of events from 5 to 6 greatly increased computational time. For these reasons, dynamic graphlets were not included in our discussion of real-world networks.

**Table 6:** Execution times of GoT with 4-nodes and DG with 4-nodes and 5 (DG-5) or 6 (DG-6) events. An asterisk (*) means that the method did not finish in the maximum running time of 1 week.

	GoT	DG-5	DG-6
Escorts	8 sec	2 hours	4 hours
Philadelphia	0.5 sec	25 hours	*
Minneapolis	2 sec	12 hours	*
Enron	2 min	1 day	4 days
Gallery	24 sec	16 hours	3 days
Escorts	8 sec	2 hours	4 hours
Transfers	3 sec	40 min	1 hours

### Network overview

A set of 14 temporal networks N was collected from various sources in order to evaluate our method’s efficiency (Table 5). N is comprised of active-edge networks, meaning that edges are only present in the snapshot *S*_*N*,*i*_ in which they appear at and need to be re-activated in subsequent snapshots. The number of snapshots $|S_{N}|$ depends on the amount of available data of *N*. Long-term networks, such as co-authorship networks, have a bigger time-interval *ρ* when compared with short-term networks, such as physical interactions in social events.

**Fig 6:**
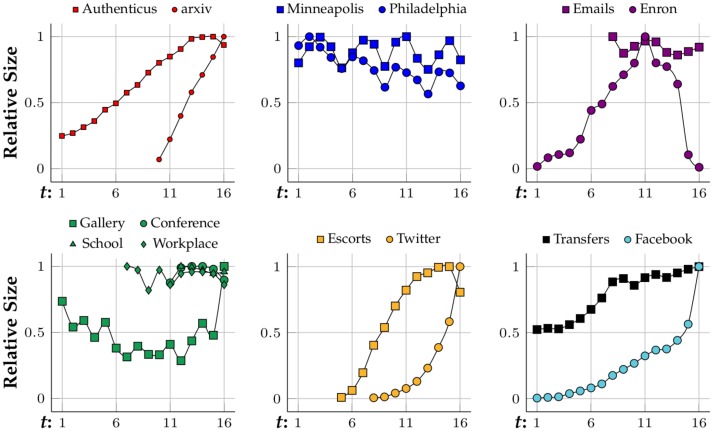
Network growth according to its number of nodes—Grouped by type.

**Fig 7:**
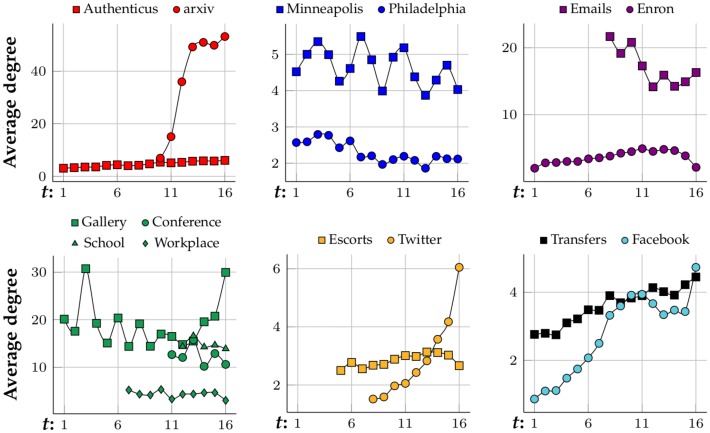
Average degree of the networks by time—Grouped by type.

**Fig 8:**
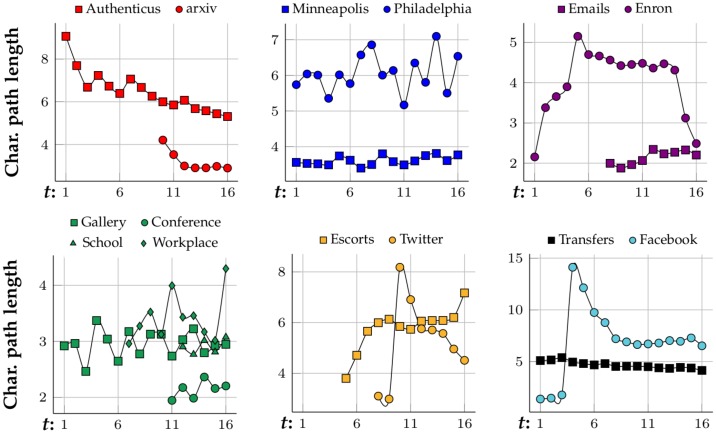
Characteristic path length of the networks by time—Grouped by type.

Fig 6 shows how the networks are evolving size-wise. Most of them are growing as time goes by. The fastest growing networks are arXiv hep-ph, Twitter, Facebook and Enron, which start at only ≈10% of their largest state, but Enron begins shrinking at *t* = 11 and almost disappears by *t* = 16. Authenticus, Escorts and Transfers are also growing networks but they grow at a slower rate and become almost stagnant at the end, where they might have reached their full potential in terms of growth. Crime, physical interaction networks and Emails stay relatively stable in size. Fig 7 presents the evolution of the networks’ average degree. arXiv hep-ph, Emails and physical interaction networks are the ones with higher average degree. arXiv hep-ph, Twitter and Facebook are the fastest growing in terms of their average degree and most networks have a stable average degree. By observing Fig 8 one can conclude that all networks from N are small-world since their characteristic-path-length at latter stages (*t* ≈ 16) is between 2 and 7. No clear correlation linking category with characteristic-path-length evolution, growth or average degree is observed from Figs 6, 7 and 8, respectively. Clustering coefficients were also computed for each network snapshot and it was found that they do not change with *t*. Co-authorship networks have the highest clustering coefficient at 0.5 while crime, bipartite, Facebook and Tranfers networks have near-zero clustering coefficient. The clustering coefficient is capable of grouping co-authorship networks together despite only considering 3-node subgraphs (triangles and 3-node chains). However, it does not distinguish between crime and bipartite networks, for instance. In these cases, one option to differentiate between networks with similar 3-node subgraphs is to analyze their 4-node network motifs and graphlets.

### Network motifs

In our experiments we performed subgraph census with *k* = 4 and *k* = 5. Results are presented only for the smaller subgraphs since no significant differences were observed. Subgraph enumeration and necessary motif statistical significance tests were performed using GT-Scanner by [30], available at http://www.dcc.fc.up.pt/~daparicio/software. Network motifs were enumerated in the final aggregate state of each network from Table 5 and motif scores Δ_*s*,*N*_ were computed for each subgraph *G*_*s*_ (Eq 1) and normalized (Eq 2). Motif fingerprints between two networks are compared by computing their Euclidean distance.

**Fig 9:**
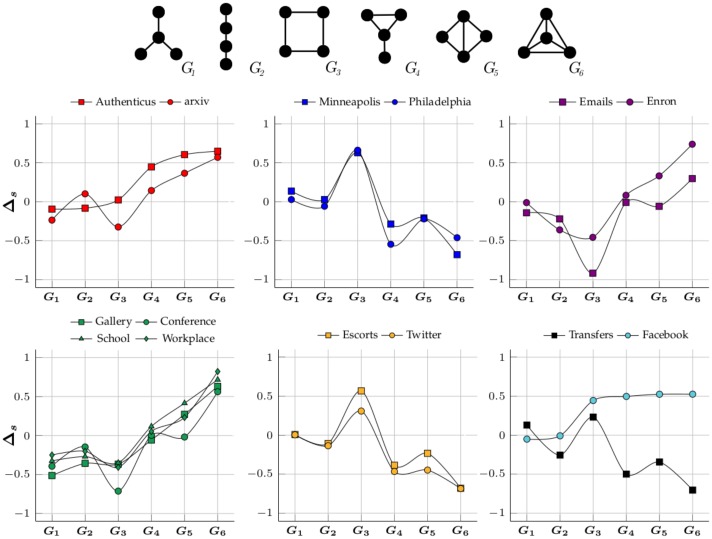
Motif-fingerprints of the networks by time—Grouped by type.

**Fig 10:**
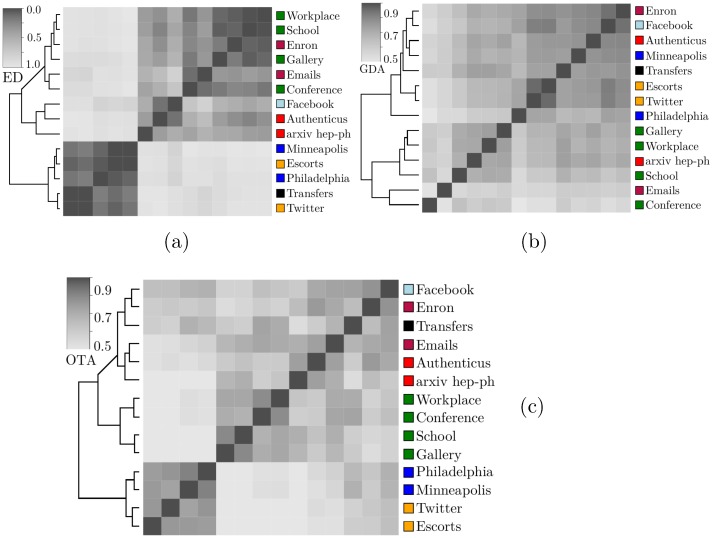
Similarity matrices according to (a) motif-fingerprints’ Euclidean distance (*ED*), (b) graphlet-degree-agreement (*GDA*) and (c) orbit-transition-agreement (*OTA*). Clustering is performed using hierarchical clustering with complete linkage.

Fig 9 shows the obtained motif-fingerprints for all 4-node undirected subgraphs ($uG_{4}$), evaluated against 100 randomized networks. Co-authorship networks have a similar motif-profile where cliques and near-cliques are the most unexpectedly prevalent groups. This comes from the fact that scientific collaboration communities tend to be tightly connected [16]. The two crime networks have a similar network profile, with cliques and near-cliques being underrepresented while squares (*G*_3_) are very overrepresented. This result was expected since our crime networks are geographical graphs with near-zero clustering coefficient and cities have a grid-like structure. Motif-profiles of the email networks are also relatively alike. Similar to co-authorship networks, cliques and near-cliques are the most overrepresented subgraphs. However, that is much more obvious in Enron than in Emails. This is probably because Emails is too small for the over-representation to become obvious since the small random networks are also capable of generating cliques and near-cliques. Physical interaction networks have a similar motif-fingerprint but it seems indistinguishable from co-authorship networks. Both types of networks have cliques and near-cliques as the most overrepresented subgraphs but those groups have different meanings. In co-authorship networks they might indicate communities but in the short-term networks they seem to simply indicate that everyone communicates with everyone by the end of the time-frame. Analyzing just the final aggregate network ignores relevant information, it is often more insightful to study how networks evolve. Bipartite networks have similar motif-fingerprints but they are also identical to those of crime networks. It should be pointed out that these networks are not pure bipartite networks but only nearly bipartite, otherwise subgraphs with cycles would never occur (*G*_3_, *G*_4_, *G*_5_ and *G*_6_). The Transfer network’s motif fingerprint is also similar to the ones of crime and bipartite networks. Finally, Facebook’s motif-profile is alike co-authorship network except *G*_3_ is also overrepresented. Since Facebook’s density is so low ($\frac{N}{E^{2}}\approx \frac{183000}{64000^{2}}\approx 0.004\%$) randomized networks have almost exclusively stars (*G*_1_) and chains (*G*_2_). Finally, by observing Fig 10(a) it is clear that motifs can only separate the networks into two big groups.

### Static graphlets

Graphlets are subgraphs that take into account the position that nodes occupy in them. Fig 1 shows set $uO_{4}$, representing all orbits of $uG_{4}$. As stated in Problem 2, graphlet-agreement computation requires graphlet-orbits to be counted for all nodes in *N*. After obtaining *GDD* matrices for all N∈N we compute the *GDA* for all network pairs. This results in a *GDA*_*i*,*j*_ matrix where *GDA*(*N*_*i*_, *N*_*j*_) ≈ 0 means that networks *N*_*i*_ and *N*_*j*_ are completely different and *GDA*(*N*_*i*_, *N*_*j*_) ≈ 1 translates to *N*_*i*_ and *N*_*j*_ being very similar. Fig 10(b) shows the obtained *GDA*_*i*,*j*_ matrix where each cell is colored according to the *GDA* value and similar networks have a darker cell. Graphlets group bipartite networks and most of the physical interactions networks correctly. By comparison, motif-fingerprints were only capable of finding two large groups, as discussed in the previous section. Neither motifs nor graphlets were able to cluster the set of networks correctly, which might indicate that temporal information is relevant to understand these networks.

### Graphlet-orbit transitions

**Fig 11:**
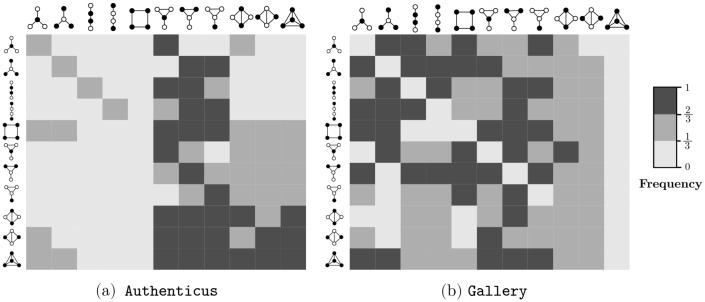
Orbit-transition matrices of (a) a collaboration network and a (b) physical interaction network for all 4-node orbits.

**Fig 12:**
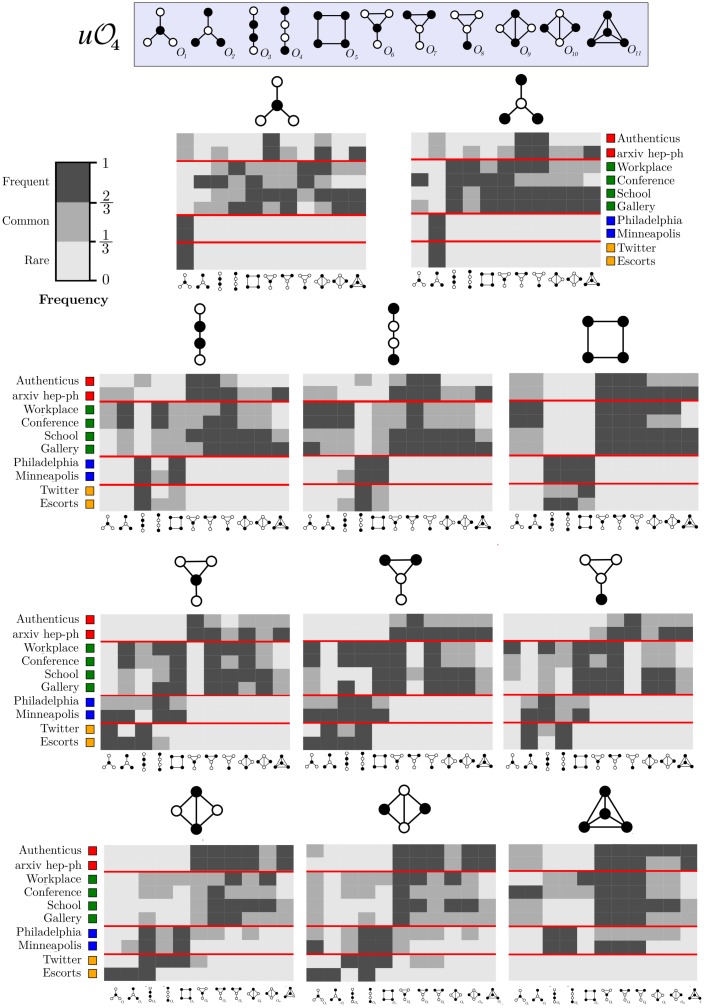
Orbit-transition fingerprints for collaboration, physical interaction, crime and bipartite networks. Frequency values are discretized into *rare*, *common* and *frequent* transitions.

All possible transitions between graphlet-orbits from the set $uO_{4}$ (Fig 1) were considered in our experiments. Enumerating larger subgraphs was unnecessary since our method already achieves an adequate grouping for *k* = 4. Furthermore, larger subgraphs would be harder to visualize in paper format. Previous studies analyzed graphlet transitions [24, 25], but graphlet-orbit transitions give more information since they account for changes of position in the same graphlet, for instance. A full orbit enumeration was performed for each snapshot *S*_*i*_ in order to build graphlet-orbit transitions matrices $uT_{4}$ for each network from Table 5. Fig 11 shows the transition matrices of Authenticus, a collaboration network, and Conference, a physical interaction network. To simplify visualization, *OTA* values were discretized into three intervals, indicating *rare* ($\left[0,\frac{1}{3}\right]$), *common* ($]\frac{1}{3},\frac{2}{3}]$) and *frequent* transitions ($]\frac{2}{3},1]$). The main diagonal of the matrix suggests that all orbits are relatively stable in Authenticus except for the square-orbit *O*_5_. This is expected from collaboration networks since groups forming a square-graph are only loosely connected, therefore these groups tend to either become tighter (transition from *O*_5_ to orbits 6-11) or nearly break apart (transition from *O*_5_ to orbits 1-4). On the other hand, orbits in Conference are very unstable, i.e. they almost always change to another orbit. This is explained by the fact that, in short-term physical interaction groups, connections are mostly temporary and not a strong indicator of community. In this example, people meet in a conference and they might meet people that their “group” already met, but they are mostly interested in meeting more people than establishing strong groups. As another example, *O*_1_ shows the effect of hubs in collaboration networks: it is more likely that a hub-like group will gain a new edge between previously unconnected authors (transition from *O*_1_ to *O*_6_) than for to remain unconnected. It is also common that not only one but two new edges appear (transition from *O*_1_ to *O*_9_). However, stars (*O*_1_/*O*_2_) becoming cliques (*O*_11_) is rare in Authenticus. Interestingly, Fig 12 shows that star-to-clique transitions are common in the other collaboration network, arXiv hep-ph. This might come from the fact that, while Authenticus data covers multiple areas, arXiv hep-ph only has publications pertaining to physicists; therefore, the observed differences may hint that physicists form tighter connections sooner than the average. It also seems that transitions are relatively slow in collaboration networks since it is rare for a loosely connected subgraph to become a densely connected subgraph in just a single jump. The same cannot be said about Conference, where behavior is almost chaotic. These are only some of the possible observations about transition matrices that highlight their interpretive power. Fig 10(c) clearly shows that graphlet-orbit transitions are able to correctly group our set of temporal networks while motifs and static graphlet-orbits could not (Fig 10(a) and 10(b)).

For completeness, Fig 12 presents orbit transitions for collaboration, physical interaction, crime and bipartite network. Matrices are discriminated by starting orbit (each matrix) and by network (each matrix-row) for an easier comparison. For instance, the first matrix from Fig 12 shows, for each network, the transitions of *O*_1_ to all $O_{k}\in uO_{4}$, the second one of *O*_2_ to all $O_{k}\in uO_{4}$, and so forth. To help visualization we inserted red lines that separate networks of different categories. It is clear that, while networks of the same category have some differences in their orbit-transition profile, they are more alike than networks from different categories. As an example: the transitions of *O*_1_ clearly distinguish co-authorship from physical interaction networks, and also co-authorship from crime and bipartite networks. However, *O*_1_ transitions are very similar for crime and bipartite networks. Distinguishing these two types of networks can be achieved by instead looking at *O*_5_, for instance. Orbit-transition fingerprints are a visual way of interpreting how a network evolves and present very detailed topological and temporal information.

## Conclusion

In this paper we put forward a new extension of graphlets for temporal networks (GoT), as well as a novel metric (OTA) to compare them. The effectiveness of our proposed method was assessed on (a) synthetic networks pertaining to well-studied graph models and (b) a set of temporal networks with predetermined categories. Our method was shown to be more accurate than competing approaches on synthetic data. For real networks, we began by analyzing how global metrics evolved over time, namely the average-degree, clustering-coefficient and the characteristic path-length. While these metrics give insight into the topological structure of the networks, we could not visualize that networks of different categories are distinguishable using them. Static network motif and graphlet analyses were also conducted since they capture richer topological information than aforementioned global metrics. However, since they do not take temporal information into account, they are not adequate for temporal network comparison. Our method correctly clustered the set of networks by category, showcasing both the importance of temporal information in these networks and our method’s clustering capabilities. Furthermore, our method produces highly interpretable results, leading to a better understanding of network evolution and differences between transitions of distinct networks.

## References

[1] Costa LdF, Oliveira ON Jr, Travieso G, Rodrigues FA, Villas Boas PR, Antiqueira L, et al Analyzing and modeling real-world phenomena with complex networks: a survey of applications. Advances in Physics. 2011;60(3):329–412. 10.1080/00018732.2011.572452

[2] Holme P, Saramäki J. Temporal networks. Physics reports. 2012;519(3):97–125. 10.1016/j.physrep.2012.03.001

[3] Milo R, Shen-Orr S, Itzkovitz S, Kashtan N, Chklovskii D, Alon U. Network motifs: simple building blocks of complex networks. Science. 2002;298(5594):824–827. 10.1126/science.298.5594.824 12399590

[4] Pržulj N. Biological network comparison using graphlet degree distribution. Bioinformatics. 2007;23:177–183. 10.1093/bioinformatics/btl301 17237089

[5] Mangan S, Alon U. Structure and function of the feed-forward loop network motif. Proceedings of the National Academy of Sciences. 2003;100(21):11980–11985. 10.1073/pnas.2133841100 PMC218699 14530388

[6] Zhu D, Qin ZS. Structural comparison of metabolic networks in selected single cell organisms. BMC bioinformatics. 2005;6(1):1. 15649332 10.1186/1471-2105-6-8 PMC549204

[7] Milo R, Itzkovitz S, Kashtan N, Levitt R, Shen-Orr S, Ayzenshtat I, et al Superfamilies of evolved and designed networks. Science. 2004;303(5663):1538–1542. 10.1126/science.1089167 15001784

[8] Faisal FE, Milenković T. Dynamic networks reveal key players in aging. Bioinformatics. 2014;30(12):1721–1729. 10.1093/bioinformatics/btu089 24554629

[9] Hulovatyy Y, Chen H, Milenković T. Exploring the structure and function of temporal networks with dynamic graphlets. Bioinformatics. 2015;31(12):i171–i180. 10.1093/bioinformatics/btv227 26072480 PMC4765862

[10] Nicosia V, Tang J, Mascolo C, Musolesi M, Russo G, Latora V. Graph metrics for temporal networks. In: Temporal networks. Springer; 2013. p. 15–40.

[11] Kelley BP, Sharan R, Karp RM, Sittler T, Root DE, Stockwell BR, et al Conserved pathways within bacteria and yeast as revealed by global protein network alignment. Proceedings of the National Academy of Sciences. 2003;100(20):11394–11399. 10.1073/pnas.1534710100 PMC208768 14504397

[12] Shah N, Koutra D, Zou T, Gallagher B, Faloutsos C. Timecrunch: Interpretable dynamic graph summarization. In: Proceedings of the 21th ACM SIGKDD International Conference on Knowledge Discovery and Data Mining. ACM; 2015. p. 1055–1064.

[13] Yu W, Aggarwal CC, Wang W. Temporally factorized network modeling for evolutionary network analysis. In: Proceedings of the Tenth ACM International Conference on Web Search and Data Mining. ACM; 2017. p. 455–464. 10.1145/3018661.3018669 PMC5470848 28626845

[14] Adhikari B, Zhang Y, Bharadwaj A, Prakash BA. Condensing temporal networks using propagation. In: Proceedings of the 2017 SIAM International Conference on Data Mining. SIAM; 2017. p. 417–425.

[15] Cook SA. The complexity of theorem-proving procedures. In: Proceedings of the third annual ACM symposium on Theory of computing. ACM; 1971. p. 151–158.

[16] Choobdar S, Ribeiro P, Bugla S, Silva F. Comparison of co-authorship networks across scientific fields using motifs. In: Advances in Social Networks Analysis and Mining (ASONAM), 2012 IEEE/ACM International Conference on. IEEE; 2012. p. 147–152.

[17] Wu G, Harrigan M, Cunningham P. Classifying Wikipedia articles using network motif counts and ratios. In: Proceedings of the Eighth Annual International Symposium on Wikis and Open Collaboration. ACM; 2012. p. 12.

[18] Buriol LS, Frahling G, Leonardi S, Marchetti-Spaccamela A, Sohler C. Counting triangles in data streams. In: Proceedings of the twenty-fifth ACM SIGMOD-SIGACT-SIGART symposium on Principles of database systems. ACM; 2006. p. 253–262.

[19] Pavan A, Tangwongsan K, Tirthapura S, Wu KL. Counting and sampling triangles from a graph stream. Proceedings of the VLDB Endowment. 2013;6(14):1870–1881. doi: 10.14778/2556549.2556569

[20] Finocchi I, Finocchi M, Fusco EG. Counting small cliques in mapreduce; 2014.

[21] Aliakbarpour M, Biswas AS, Gouleakis T, Peebles J, Rubinfeld R, Yodpinyanee A. Sublinear-time algorithms for counting star subgraphs with applications to join selectivity estimation. arXiv preprint arXiv:160104233. 2016;.

[22] Kovanen L, Karsai M, Kaski K, Kertész J, Saramäki J. Temporal motifs in time-dependent networks. Journal of Statistical Mechanics: Theory and Experiment. 2011;2011(11):P11005 10.1088/1742-5468/2011/11/P11005

[23] Martin AJ, Dominguez C, Contreras-Riquelme S, Holmes DS, Perez-Acle T. Graphlet Based Metrics for the Comparison of Gene Regulatory Networks. PloS one. 2016;11(10):e0163497 10.1371/journal.pone.0163497 27695050 PMC5047442

[24] Doroud M, Bhattacharyya P, Wu SF, Felmlee D; IEEE. The evolution of ego-centric triads: A microscopic approach toward predicting macroscopic network properties. 2011; p. 172–179.

[25] Kim MS, Kim JR, Kim D, Lander AD, Cho KH. Spatiotemporal network motif reveals the biological traits of developmental gene regulatory networks in Drosophila melanogaster. BMC systems biology. 2012;6(1):31 10.1186/1752-0509-6-31 22548745 PMC3434043

[26] Jin R, McCallen S, Almaas E. Trend motif: A graph mining approach for analysis of dynamic complex networks. In: Seventh IEEE International Conference on Data Mining (ICDM 2007). IEEE; 2007. p. 541–546.

[27] Kobler J, Schöning U, Torán J. The graph isomorphism problem: its structural complexity. Springer Science & Business Media; 2012.

[28] McKay B, Piperno A. Practical graph isomorphism. Journal of Symbolic Computation. 2014;60(0):94–112. 10.1016/j.jsc.2013.09.003

[29] Wasserman S, Faust K. Social network analysis: Methods and applications. vol. 8 Cambridge university press; 1994.

[30] Aparicio D, Ribeiro P, Silva F. Extending the Applicability of Graphlets to Directed Networks. IEEE/ACM Transactions of Computational Biology and Bioinformatics. 2016;PP. 10.1109/TCBB.2016.2586046 27362986

[31] Milenković T, Lai J, Pržulj N. GraphCrunch: a tool for large network analyses. BMC bioinformatics. 2008;9(1):70 10.1186/1471-2105-9-70 18230190 PMC2275247

[32] Hočevar T, Demšar J. A combinatorial approach to graphlet counting. Bioinformatics. 2014;30(4):559–565. 10.1093/bioinformatics/btt717 24336411

[33] Ribeiro P, Silva F. G-Tries: a data structure for storing and finding subgraphs. Data Mining and Knowledge Discovery. 2014;28(2):337–377. 10.1007/s10618-013-0303-4

[34] Erdös P, Rényi A. On the evolution of random graphs. Publ Math Inst Hung Acad Sci. 1960;5(17-61):43.

[35] Barabási AL, Albert R. Emergence of scaling in random networks. science. 1999;286(5439):509–512. 10.1126/science.286.5439.509 10521342

[36] Watts DJ, Strogatz SH. Collective dynamics of small-world networks. nature. 1998;393(6684):440–442. 10.1038/30918 9623998

[37] Leskovec J, Backstrom L, Kumar R, Tomkins A. Microscopic evolution of social networks. In: Proceedings of the 14th ACM SIGKDD international conference on Knowledge discovery and data mining. ACM; 2008. p. 462–470.

[38] Hu H, Wang X. Evolution of a large online social network. Physics Letters A. 2009;373(12):1105–1110. 10.1016/j.physleta.2009.02.004

[39] Paredes P, Ribeiro P. Towards a faster network-centric subgraph census. In: Advances in Social Networks Analysis and Mining (ASONAM), 2013 IEEE/ACM International Conference on. IEEE; 2013. p. 264–271.

[40] Ribeiro P, Silva F. Efficient subgraph frequency estimation with g-tries. In: International Workshop on Algorithms in Bioinformatics. Springer; 2010. p. 238–249.

[41] Aparício DO, Ribeiro PMP, da Silva FMA. Parallel subgraph counting for multicore architectures. In: Parallel and Distributed Processing with Applications (ISPA), 2014 IEEE International Symposium on. IEEE; 2014. p. 34–41.

[42] Pinar A, Seshadhri C, Vishal V. Escape: Efficiently counting all 5-vertex subgraphs. In: Proceedings of the 26th International Conference on World Wide Web. International World Wide Web Conferences Steering Committee; 2017. p. 1431–1440.

[43] Leskovec J, Kleinberg J, Faloutsos C. Graph evolution: Densification and shrinking diameters. ACM Transactions on Knowledge Discovery from Data (TKDD). 2007;1(1):2 10.1145/1217299.1217301

[44] Risdal M. Minneapolis Incidents & Crime; 2018. https://www.kaggle.com/mrisdal/minneapolis-incidents-crime.

[45] Chirico M. Phildelphia Crime Data; 2018. https://www.kaggle.com/mchirico/philadelphiacrimedata.

[46] Michalski R, Palus S, Kazienko P. Matching Organizational Structure and Social Network Extracted from Email Communication. In: Lecture Notes in Business Information Processing. vol. 87. Springer Berlin Heidelberg; 2011. p. 197–206.

[47] Leskovec J, Lang KJ, Dasgupta A, Mahoney MW. Community structure in large networks: Natural cluster sizes and the absence of large well-defined clusters. Internet Mathematics. 2009;6(1):29–123. 10.1080/15427951.2009.10129177

[48] Isella L, Stehlé J, Barrat A, Cattuto C, Pinton JF, Van den Broeck W. What’s in a crowd? Analysis of face-to-face behavioral networks. Journal of theoretical biology. 2011;271(1):166–180. 10.1016/j.jtbi.2010.11.033 21130777

[49] Stehlé J, Voirin N, Barrat A, Cattuto C, Isella L, Pinton JF, et al High-resolution measurements of face-to-face contact patterns in a primary school. PloS one. 2011;6(8):e23176 10.1371/journal.pone.0023176 21858018 PMC3156713

[50] Génois M, Vestergaard CL, Fournet J, Panisson A, Bonmarin I, Barrat A. Data on face-to-face contacts in an office building suggest a low-cost vaccination strategy based on community linkers. Network Science. 2015;3(3):326–347. 10.1017/nws.2015.10

[51] Rocha LE, Liljeros F, Holme P. Information dynamics shape the sexual networks of Internet-mediated prostitution. Proceedings of the National Academy of Sciences. 2010;107(13):5706–5711. 10.1073/pnas.0914080107 PMC2851932 20231480

[52] Choudhury MD, Lin YR, Sundaram H, Candan KS, Xie L, Kelliher A. How Does the Data Sampling Strategy Impact the Discovery of Information Diffusion in Social Media? In: ICWSM; 2010. p. 34–41.

[53] Viswanath B, Mislove A, Cha M, Gummadi KP. On the Evolution of User Interaction in Facebook. In: Proc. Workshop on Online Social Networks; 2009. p. 37–42.

